# The Trend in Mitigation Strategies of 3-Monochloropropane-1,2-diol and Glycidyl Esters in Edible Vegetable Oils: Today and Tomorrow

**DOI:** 10.17113/ftb.62.03.24.8260

**Published:** 2024-09

**Authors:** Kıvılcım Yıldız, Onur Özdikicierler, Pelin Günç Ergönül

**Affiliations:** 1 Manisa Celal Bayar University, Engineering Faculty, Department of Food Engineering, Prof. Dr. Ilhan Varank Campus, 45030 Manisa, Türkiye; 2 Ege University, Engineering Faculty, Department of Food Engineering, 35050 İzmir, Türkiye

**Keywords:** mitigation of 3-MCPD and GE, detection in edible oils, genotoxic and carcinogenic contaminants, reduction of precursor formation

## Abstract

3-Monochloropropane-1,2-diol (3-MCPD) and its esters, which have carcinogenic and genotoxic effects, are contaminants induced by high-temperature that have been detected in refined oils and fatty foods. 3-MCPD esters, chlorinated propanols, were first identified in 1978 in acid-hydrolysed vegetable proteins used as flavour enhancers in many foods. Glycidyl esters (GE) are contaminants that can occur in edible oils during heat treatment and are formed mainly during the deodorisation phase of refining. The International Agency for Research on Cancer has classified 3-MCPD as a ’potential carcinogen for humans’ in group 2B. Glycidol has also been classified as group 2A with mutagenic and carcinogenic properties, *i.e.* ’probably carcinogenic to humans’. In addition, glycidol has been classified by the International Agency for Research on Cancer (IARC) as a ’possible human carcinogen’ (group 2A). Toxicological studies have shown that these genotoxic and carcinogenic contaminants induced by heat treatment are released in the gastrointestinal tract and cause the formation of tumours. In this review the mechanisms of formation, toxicological effects of 3-MCPD and GE on human health, and methods of their detection are shown. The latest strategies to mitigate and prevent 3-MCPD and GE formation during crude oil production, refining and beyond are also discussed.

## INTRODUCTION

Fats and oils are macronutrients that can be consumed directly or used in the preparation of food (*1*–*3*). Today, vegetable oils constitute 80 % of the fat and oil production worldwide (*2*, *4*). Edible oils are obtained from plant seeds or pulp by mechanical, thermal or chemical methods (*2*). Some foods undergo heat treatment during production, which can lead to the formation of contaminants that can have toxic effects on the consumers (*5*). Fats and oils are refined to improve their sensory properties and consumer acceptance (*2*, *6*). Since higher temperatures are applied during these refining steps, the process temperature is a decisive factor for the sensory, physical and chemical properties of the refined oils (*7*).

During oil refining, free fatty acids and minor contaminants such as pesticides and polyaromatic hydrocarbons, as well as undesirable flavour and colour, are removed or reduced (*6*, *8*). As a side effect, undesirable compounds (*trans* fatty acids, acylglycerol polymers, cyclic fatty acid esters and 3-MCPDE) can be formed in oils at high temperatures used for oil refining (*9*–*12*). 3-MCPDE and GE, which have carcinogenic and genotoxic effects, are among the contaminants detected in refined oils (especially palm oil) and fatty foods (*13*–*19*). 3-MCPDE consists of acylglycerols released at temperatures above 200 °C in the presence of organic or inorganic chlorinated compounds (*2*). These compounds then react with chloride ions and are converted into 3-MCPDE (*20*).

3-MCPDE and GE are formed in vegetable oils especially during the deodorisation step, when their amount highly increases (*3*, *17*, *21*-*23*). They are relatively higher in palm oil than in refined vegetable oils such as soybean, canola and sunflower (*16*, *24*–*26*).

Studies have shown that chloropropanols can be formed both in ester form (linked) and in free form (*8*, *23*). The free form of 3-MCPDE was first identified by Velíšek *et al.* in acid-hydrolysed vegetable proteins, which are used as flavour enhancers in many foods (*14*, *16*, *24*). It was first discovered in 1980 by Davidek *et al.* (*27*) in the ester form. In 2001, the European Commission Scientific Committee on Food (SCF) set the maximum content for 3-MCPD in hydrolysed vegetable protein (HVP) and soy sauce at 20 µg/kg (*28*). In 2006, Zelinková *et al.* (*29*) found that vegetable fats and oils contain high amounts of 3-MCPDE formed during refining. Later, the tolerable daily intake was set by JECFA (FAO/WHO Joint Experts Committee on Food Additives) and Scientific Committee on Food at 2 µg per kg of body mass (*25*).

The latest European Commission Regulation set the limit value for GE in vegetable oils at 1 mg/kg (*26*). Over time, these contaminants have been detected in most processed foods such as meat, milk, grain products, malt products, coffee, fried cheese, smoked foods and biscuits (*2*, *8*, *13*, *15*, *17*, *28*, *29*). The study carried out by Wöhrlin *et al.* (*30*) showed that 3-MCPDEs can even be found in infant food formula. It is confirmed that high amounts of 3-MCPDE in foods, especially in refined vegetable oils and fats, can cause health problems due to their toxicity in the free form (*3*). According to the report of The German Federal Institute for Risk Assessment (BfR), 3-MCPD is completely degraded from its esters in the gastrointestinal tract (*3*). Some studies have also shown that these compounds change from ester to free forms (*17*, *31*, *32*). In addition, studies by Abraham *et al.* (*33*) and Andres *et al.* (*34*) reported that the relative bioavailability of 3-MCPDE was 86 %. According to the Joint FAO/WHO Expert Committee on Food Additives (JECFA), the main target organ of 3-MCPD toxicity is the kidneys, causing nephropathy, tubular hyperplasia and adenomas (*35*, *36*). The International Agency for Research on Cancer (IARC) classifies 3-MCPDE as carcinogenic compounds (*31*, *32*, *37*, *38*). Studies carried out on experimental mice have shown that 3-MCPDE in free form have nephrotoxic effects and cause the formation of tumours. The International Agency for Research on Cancer classified 3-MCPD as a ’potential carcinogen for humans’ in group 2B (*39*). On the other hand, glycidol with mutagenic and carcinogenic properties was classified in group 2A and labelled as ’most likely carcinogenic to humans’ (*17*, *40*–*42*).

**Fig. 1:**
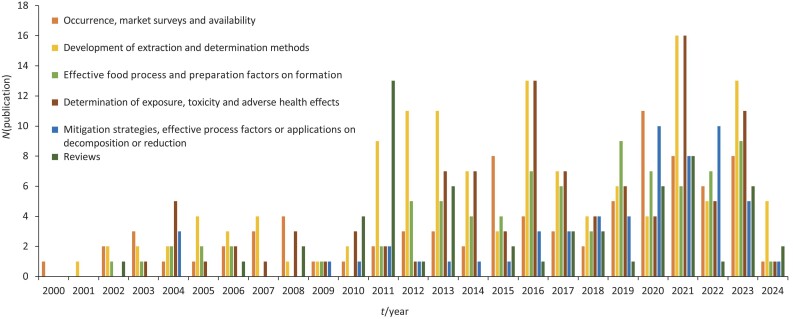
Distribution of published scientific articles indexed in different categories in the Web of Science database between 2000 and 2024

In the last decade, scientific interest in MCPDE and GE has increased. A literature search was carried out in the Web of Science database using the keywords ’MCPD’ and ’glycidyl esters’ for the period between 2000 and 2024. The results were sorted according to the relevance of the search criteria. Those related to disciplines other than food technology were excluded. If the proceedings are ignored, access to 529 distinct published journal articles is possible. The published articles were categorised into five main topics, as shown in Fig. 1. The topic that received the most attention was the toxicity, bioavailability and adverse health effects of 3-MCPD and GE. In addition, several articles focused on the methods of analysis and validation. Since 2011, the number of publications has increased significantly, and it stands out as the year with the highest number of published review papers. The blue bar shows the number of studies published on the mitigation of 3-MCPD and GE in oils and fats. Among the newly developed methods, the combination of ultrasound to accelerate the esterification part and n-hepta-fluorobutyrylimidazole (HFBI) derivatisation during dispersive liquid-liquid microextraction (DLLME) has a positive effect on both cost and environmental friendliness (*43*).

Foods and refined vegetable oils containing 3-MCPD and its esters, which have carcinogenic and mutagenic effects, can be consumed directly. International committees and studies have demonstrated the adverse effects of these contaminants on health. For this reason, it is crucial to prevent the formation of 3-MCPD and its esters in refined vegetable oils and foods or to reduce their amounts. Unlike other studies on this topic, this review provides detailed data on the formation of 3-MCPDE and their toxic effects, analytical extraction and determination methods, reduction strategies, preventive/reduction procedures that can be applied in the extraction and refining of oils and fats, and current regulations. Even though we found 529 articles in the literature search, we only selected and summarised recently published studies on specific topics. While we have primarily summarised recently published studies, we have also cited older, important studies that provide relevant background information on the topic.

## TOXICITY OF 3-MCPD AND GE, AND RELATED REGULATIONS

**Fig. 2:**
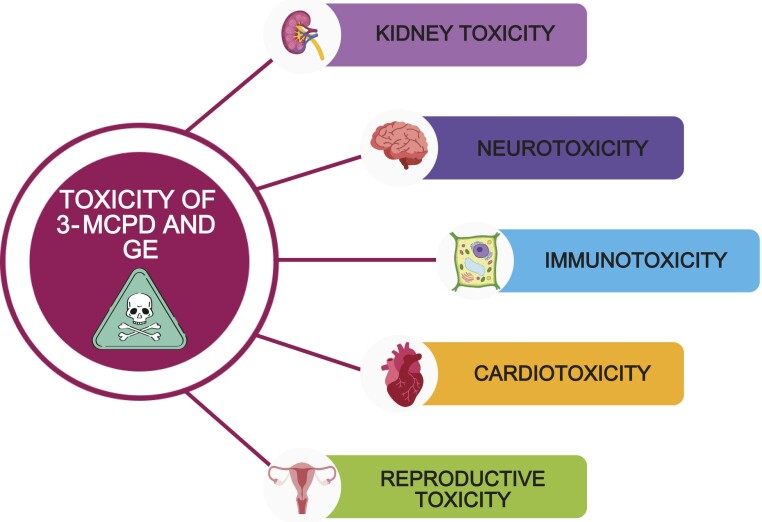
Toxicity of 3-monochloropropane-1,2-diol (3-MCPD) and glycidyl ester (GE)

3-Monochloropropane-1,2-diol (3-MCPD) and (2,3-epoxy-1-propanol) glycidol and their esters (3-MCPDE and GE), contaminants in food processing that are harmful to human health, are formed during the refining of vegetable fats and oils, especially during deodorisation at high temperatures. Previous studies have shown that these components can be found in baby food, margarine, coffee, soy sauce, bakery products, soup, meat, milk, malt products, fried cheese and smoked foods. 3-MCPD and GE have been declared as ingredients that jeopardise food safety worldwide and cause numerous health problems. Studies have shown that 3-MCPD, when consumed regularly in substantial amounts, impairs male fertility, kidney function and body mass of rats (Fig. 2) (*3*, *44*–*46*). 1,3-DCP, the ester of 3-MCPD, has been found to be a genotoxic, hepatotoxic and carcinogenic substance that is classified as a human carcinogen (*46*). The International Agency for Research on Cancer (IARC) has defined 3-MCPD as a group 2B ’substance with possible carcinogenic effects on humans’ and GE as a group 2A ’substance with possible carcinogenic effects on humans’ (*47*). Studies on the potential toxic effects of 3-MCPDE and GE have not yet been completed and many unknowns on this topic remain. Various toxicological studies have reported that long-term consumption of high doses of these compounds leads to nephritic and reproductive organ failure and hyperplasia in particular (*3*, *25*, *33*, *34*, *36*, *48*–*50*). In a study on mice, neurotoxic effects such as paralysis were observed after short-term exposure to a high dose of 50 mg/kg per day (*49*). Almost all 3-MCPDE have been reported to convert to the free form under the influence of lipase in the gastrointestinal tract (GIT), which causes toxicity (*34*, *51*–*55*). Similarly, GE are predominantly hydrolysed to the genotoxic carcinogen glycidol, which can mediate detrimental effects in the gastrointestinal tract (*21*, *55*). In addition, 3-MCPD has been found to cause severe proteinuria and glycosuria as well as acute glomerulonephritis, renal and testicular tumourigenesis in mice (*56*–*59*). Studies on mice have shown that free 3-MCPD is not absorbed by intestinal cells but passes through the cell surface and causes renal tubular carcinoma and Leydig cell tumour formation in mature male rats, effectively in the kidney and reproductive system (*34*, *48*, *52*). One of the potential toxicity mechanisms of 3-MCPD is its oxidative metabolism to chlorolactaldehyde and chlorolactic acid, which causes oxidative stress and disrupts the glycolytic pathway and energy production (*60*). GE, which are listed in group 2A, have been classified as genotoxic compounds in *in vivo* and *in vitro* studies (*21*, *42*, *49*). In a study in rats, glycidol administered at a dose of 200 mg/kg per day for 28 days did not cause neurotoxicity but it was reported to cause renal toxicity at repeated doses of 150–400 mg/kg (*61*–*63*). Even the lowest dose of GE exposure affected the reproductive organs in rats, and EFSA reported that daily intake of 25 mg/kg caused a 36 % decrease in sperm count (*49*). Another toxicological study in rats revealed that exposure to 3-MCPD and GE caused lung and heart toxicity. It has also been reported that exposure to glycidol can lead to ovarian dysfunction, colon disease and several carcinomas (*55*). Another study also reported that lung lesions, including alveolar oedema and hyperaemia or haemorrhagic areas, were observed in Swiss mice exposed to 3-MCPD (*36*).

The report published by EFSA (*49*) in 2016 includes the chemical structures, formation mechanisms, amounts in different foods, direct and indirect analysis methods and toxicological properties of 3-MCPD and GE. EFSA has set the maximum content of 3-MCPD in soy sauce and hydrolysed vegetable proteins at 20 µg/kg (*25*). Additionally, the JECFA set the daily tolerable intake of 3-MCPD amount per body mass at 4 mg/kg (*35*). Due to the carcinogenic and genotoxic effects of glycidol, EFSA has recommended the application of a ’limit/margin of exposure (MoE)’ approach for glycidyl esters and reported that foods with MoE greater than 25 000 should be considered risky. In this context, the regulation was amended in 2018 to specify that the GE value in vegetable oils and baby/child food should not exceed 1000 µg/kg (*25*, *49*).

Moreover, the European Union published a new regulation effective on 1 January 2021 (*64*). According to this regulation, the maximum amount of 3-MCPD and GE in oils should be 1.25 and 1 mg/kg, respectively. It was emphasised that the mass fractions of 3-MCPD and GE in baby products should not exceed 0.75 and 0.5 mg/kg, respectively. It has been reported that maximum amounts of 3-MCPD and GE in infant formula, namely in follow-on milk in liquid form should not exceed 0.125 and 0.05 mg/kg, and in follow-on milk in powder form should not exceed 0.015 and 0.006 mg/kg, respectively (*64*).

## THE EFFECTS OF THE PRECURSORS ON THE FORMATION OF 3-MCPDE AND GE

It has been reported that for the formation of 3-MCPD and esters, hydrolysed triacylglycerols (TAG) should be primarily degraded into glycerol, mono- and diacylglycerols (MAG/DAG) and then converted to MCPD in the presence of chloride ions of glycidyl esters (GE) together with the cyclic acyloxonium molecule, which is formed from these substances at high temperature (*57*, *65*). The studies and the International Life Sciences Institute (ILSI) identified MAG, DAG and chloride ions as precursors for the formation of these compounds and determined the process temperature and duration as the effective parameters for the formation of these compounds (*21*, *66*–*78*). In edible vegetable oils, MAG and DAG are formed during maturation, harvesting or transportation of oily fruit seeds from TAGs by lipase enzyme activity, and they are also formed during high-temperature deodorisation phase during oil refining (*72*, *73*). Therefore, DAG and MAG contents in different oils or the same oils obtained from different locations may also differ (*41*). Fruit oils are more sensitive to hydrolysis reactions than seed oils due to their excess water. Hence, DAG content in the fruit oils is higher than in the seed oils. Palm oil obtained from palm fruits, for example, can have a DAG content between 4 and 12 % (*74*). High rates of MAG and DAG, which are formed due to the hydrolysis reaction, lead to the formation of 3-MCPD and GE (*75*). In their study, Zelinková *et al.* (*29*) suggested that there could be a correlation between the DAG content and the MCPD amounts in refined edible oils. The reason for this is that the highest MCPD contents were found in fruit oils such as olive and palm oil. It is known that fruit extract oils have a higher DAG content than seed oils due to the faster formation of lipolytic reactions during harvesting (*77*, *78*). The studies have shown that DAGs, precursors of 3-MCPD and GE, have a stronger effect than MAGs under deodorising conditions. Compared to the same amount of mono- and diglycerides, the formation of 3-MCPD was found to be about twice as high as that of diglycerides (*5*, *18*). Another study found that the GE content in oils with high DAG content was 12 to 43 times higher than that in edible oils containing mainly TAGs (*79*). The formation of GE is directly related to high temperatures and the contact time at these temperatures, and they can be formed from diacylglycerols without requiring the presence of chlorinated compounds. In addition, the highest GE content was found in refined palm oil compared to other vegetable oils (*20*, *77*). Masukawa *et al.* (*79*) found that the GE content was 10 times higher in refined commercial oils with high DAG content (87 %) than in oils with low DAG content (3.9–6.8 %). In addition, Destaillats *et al.* (*80*) found that both MAG and DAG can cause the formation of GE during thermal treatment. It has been reported that GE is mainly present in refined palm oil and palm-based fractions and the GE content increases in direct proportion to the duration and temperature of the deodorisation process (*6*, *20*, *29*).

3-MCPD is formed by the reaction between chloride and lipid sources. This chloride source can either be the food itself or the material that comes into contact with it, which can be chlorinated water or additional salt during production (*18*, *34*, *81*-*85*). The mineral chlorine is abundant in nature. It is known that organic and inorganic chloride sources are the potential precursor compounds in the formation of MCPD. Vegetable oils contain chloride compounds in addition to TAG, DAG, MAG and free fatty acids. Destaillats *et al.* (*80*) determined that many inorganic chlorine sources were found in higher amounts (mg/kg) in crude palm oil than in other vegetable oils. Chlorides from formed during crude oil pressing and extraction, and free hydrogen chlorides from the bleaching earth pass into the oil (*86*). The type, quality, composition and production conditions of the crude oil also affect the chlorine quantity, as do MAGs and DAGs (*87*–*89*). The interaction of acylglycerols with chlorine has been reported to cause the formation of 3-MCPDE during the refining of edible oils, especially in the deodorisation step (*29*, *74*, *80*, *82*, *89*-*91*). In addition to the effects of chlorine and MAG/DAG, the processing temperature and duration are other factors that influence the mechanism of the formation of 3-MCPD esters in food (*57*, *65*, *68*). Studies have shown that the content of contaminants increase rapidly at temperatures above 200 °C, but a balance between their formation and breakdown is reached with increasing processing time (*77*, *78*, *90*-*93*). GE are unstable molecules, but 3-MCPDs are stable. After the 3-MCPD compounds are formed, their stability depends on the pH and ambient temperature. The formation of 3-MCPD increases with higher pH and temperature. The concentration of 3-MCPDE in vegetable oils depends on their composition and deodorisation. In the study conducted by Franke *et al.* (*89*) on canola oil, the 3-MCPD content in oil containing only 2 % DAG and 0.1 % free fatty acids increased from 0.4 to 1.0 mg/kg during deodorisation. In palm oil with 5 % DAG and 0.24 % free fatty acids, this rate increased from 1.0 to 4.4 mg/kg (*89*). On the other hand, Shimizu *et al.* (*72*) subjected tri-, di- and monooleins to a temperature of 240 °C in the presence and absence of chloride ions to determine the effect of the precursors that trigger the formation of 3-MCPD and GE. They found that 3-MCPDE formed from mono- and dioleins in the presence of chloride ions, while GE formed in the absence of chloride ions. The study showed that di- and monoolein are effective precursors of 3-MCPD and GE in addition to the chloride ion. It was also observed that triolein remained stable during the temperature test and caused the formation of a very small amount of esters (*72*). In another study, it was found that ester formation was higher at temperatures above 229–240 °C (*80*).

## THE STRATEGIES FOR MITIGATION OF 3-MCPD AND GE

### Changes due to the oil extraction parameters

The type of oil (the presence of glycerol, mono-, di- and triacylglycerols), oil extraction method (temperature and time) and the amount of chlorine and water in the oil are the main factors influencing the formation of 3-MCPD and GE (*71*, *93*). The extraction of the oil from fruits with a higher water content than from the seeds and the temperature during the extraction process increase the formation of contaminants (*78*, *79*). There are three different ways to reduce the amount of esters in the oils to be refined. The first way is to avoid and/or minimise precursors in the raw material, the second is to adjust and or optimise the parameters of the refining process and the third is to remove the esters formed in the oil after refining (*74*). The most important thing is to minimise the amount of precursors in the raw materials. In this way, the ester formation can be kept to a minimum. The reduction of precursors found in the raw material extends to the field, and factors such as climate, soil and growing conditions are effective. Subsequent harvesting practices and storage conditions are also important parameters for the formation of these precursors (*31*, *74*, *94*, *95*). These different conditions affect the amount of chlorine-containing compounds, which is an important precursor (*96*). Saline soils, iron(III) chloride and fertilisers (KCl and NH_4_Cl) used in water treatment are among the inorganic sources of chloride (*97*). In addition, some organochlorine pesticides used to control weeds and pests during cultivation lead to an increase in chlorine amounts in the raw material (*74*). For this reason, it is recommended to wash the palm fruits with slightly alkaline water to reduce the chlorine content in the product. Studies have shown that there is no difference in the formation of contaminants between the chlorine sources, but that the amount of 3-MCPD increases as the amount of chlorine increases (*80*, *84*, *90*). Storage under unsuitable conditions after harvest also leads to an increase in these contaminants. In oily fruits and seeds, the triglycerides are broken down by lipase into free fatty acids and diglycerides. If lipase activity is high in ripe fruit, this leads to a rapid degradation of triacylglycerols causng the formation of large amounts of diacylglycerol, which also increases the amount of formed esters (*21*, *71*, *74*, *89*). During the processing of palm fruit bunches into crude palm oil, chlorine catalyses the formation of partial acylglycerols and free fatty acids through lipolytic activity (*98*). For this reason, palm fruit bunches should be collected quickly after harvest to ensure inactivation of the enzymes, and sterilised without steam. Low temperatures should be used for sterilisation (*31*, *96*). Extraction within 48 h of harvest reduced the amount of mono- and diacylglycerols in the oil and resulted in its good quality. In addition, damaged fruits are not processed together with other products to prevent the formation of esters (*31*).

According to all this knowledge, in order to reduce the formation of 3-MCPDE, either the chlorine-containing compounds in the fruits should be reduced or these compounds should be removed before refining (*74*). Chloride-containing compounds can be removed by washing the crude palm oil with polar solvents such as water or water/alcohol mixture. Another efficient way is to wash the oilcake during oil extraction process and then remove chlorine compounds (*95*, *99*). Some researchers found that washing crude palm oil with water or water/alcohol mixture reduces 3-MCPD and GE contents (*74*, *95*).

### Reduction of precursors before processing

As mentioned before, compounds acting as a precursor in the formation of 3-MCPD and GE are MAG/DAG and chloride ions. Elimination of these compounds from the crude vegetable oils before refining will also affect the quantity of 3-MCPD and GE in the end product. The precursors of 3-MCPD and GE in the oil are directly affected by climate, soil, fertilisation, harvesting and storage conditions (*95*). Chlorinated compounds in the raw material are the most important precursors that play a role in the formation of 3-MCPDE (*96*). Many researchers have associated them with the use of fertilisers such as ammonium chloride and potassium chloride, and with the presence of chlorine in the irrigation water. The chlorine amount in the raw material depends on the variation, type of soil, growth conditions and differences in the plant genotypes (*100*). Chlorine-containing chemicals in raw materials can be reduced by using fewer or no pesticides and fertilisers (*101*). For example, conventional palm production, where fertilisers and pesticides are used that can degrade soil and leave residues, can be replaced with organic palm production without the use of fertilizers, favouring locally adapted systems over synthetic materials (*102*). Numerous chlorine compounds, including magnesium chloride, calcium chloride, iron(III) chloride and iron(II) chloride, are produced during irrigation and fertilisation (*103*). Matthäus *et al.* (*95*) found that washing crude palm oil with water or ethanol reduced the amount of 3-MCPDE by 20–30 %. Also, palm fruit pulp washing before oil extraction reduced 3-MCDPE content by 95 % compared to deodorisation. Yung *et al.* (*104*) focused on optimising the removal of chloride from crude palm oil by washing with water to minimise the production of 3-MCPDE. By adding 5 % water per oil mass during the washing process, the water-soluble chlorides in crude palm oil are eliminated by up to 76 %, resulting in a 71 % decrease in 3-MCPDE amounts and bringing them below the legal limits. A linear relationship was established between the chloride and the equivalent 3-MCPDE, with a high correlation value (R^2^) of 0.99. Using the correlations, it is possible to extract 1.0 mg/kg of 3-MCPDE from refined, bleached and deodorised palm oil obtained from crude palm oil with 1.2 mg/kg chloride, using 7 % water during washing. The study also showed a slight decrease in GE of 7 to 11 % after washing with water (*104*). Ramli *et al.* (*105*) conducted a larger scale study to reduce 3-MCPDE in palm oil. The study included washing 900 tonnes of crude palm oil in 5–10 % hot water at a temperature of 90–95 °C. Although washing did not have a statistically significant effect (p>0.05) on other oil quality indicators, such as the concentration of free fatty acids (FFA), diacylglycerol (DAG) and degradation of bleachability index (DOBI), a simple hot water washing removed more than 85 % of the total chlorine. The study found that the most effective method to produce refined oil of excellent quality with significantly low content of 3-MCPDE was water washing of crude palm oil both before and after the refining process (*105*). In another study, washing the crude palm oil with *V*(ethanol):*V*(water)=50 % before deodorisation reduced the 3-MCPDE content by 30 % (*97*). Considering all the studies conducted in this framework, it is found that washing a crude vegetable oil with polar solvents helps to remove water-soluble chlorine and reduce the formation of 3-MCPDE (*94*). In addition, previous research was conducted to reduce 3-MCPDE. The organochlorine components were removed using monoacylglycerols as agents in various oils without the use of a solvent. The study was carried out by mixing and homogenising a crude vegetable oil with monoacylglycerols and separating these components from the oil by crystallisation. Finally, the amount of 3-MCPDE was reduced, as a result of the reaction of the organochlorine compounds with monoacylglycerols, by 60–90 %, depending on the types of oils and the thermal treatment (*106*).

Unlike 3-MCPDE, the precursors of GE are MAG and DAG, but not chloride ions (*95*). The studies show that the content of MAG and DAG in crude vegetable oils has a significant effect on the formation of GE. Thus, reducing the formation of GE is considered the most effective method to reduce the amounts of MAG and DAG in vegetable oils. The amount of MAG and DAG in vegetable oils increases due to the hydrolysis of TAGs by lipase enzyme activity (*21*). Mishandling during harvesting or storage of the oily fruits or seeds causes the product damage and increased lipase activity, which results in a higher MAG and DAG content. Thus, fruits and seeds should be sterilised after harvest to inactivate lipase. However, it is recommended to keep the sterilisation temperature below 120 °C to avoid triggering the formation of MCPDE (*28*, *41*, *90*, *99*). For oil extraction after harvesting, processing fruits under unfavourable conditions also increases the amount of MAG and DAG in the oil (*74*, *107*). The location of the growth also influences the content of 3-MCPD and GE in oily seeds or fruits. The amount of 3-MCPD in oil is usually determined by the concentration of the precursor in raw material before processing (*103*).

### Strategies applicable during the refining

Vegetable oil can be refined in two ways, namely physical and chemical refining. In both cases, the main purpose of refining is to remove or reduce unwanted/undesirable ingredients. Degumming, bleaching and deodorisation are the main steps in both refining methods. Neutralisation, which is achieved by saponification of free fatty acids and separation of the aqueous soap-stock phase, is the main difference between chemical and physical refining. The refining steps were further investigated to reduce the 3-MCPDE and GE content in vegetable oil and were generally associated with optimisation approaches. Within this framework, some studies aimed to reduce the 3-MCPDE and GE content by reducing the formation rate through optimisation of the parameters of the refining steps, while others aimed to apply new steps within the refining process (Table 1; *5,20,29,37,38,72,89,108-120*).

**Table 1:** Brief information about some studies discussing mitigation of 3-monochloropropane-1,2-diol (3-MCPD) and glycidyl ester (GE) during refining steps

Oil type	Operation/step	Condition	Effect	Reference
Crude palm oil	Prewashing (applied prior to degumming)	20 min at 70 °C, 10 % deionised water	At the end of the physical refining, 2- and 3-MCPDE (*w*=0.54 and 1.04 mg/kg, respectively) were 19 and 22 % lower when prewashing was applied	(*108*)
Crude palm oil	Neutralisation	20 min at 70 °C with 2 L of 33 % NaOH solution	*w*(3-MCPDE) was reduced by 43 % but *w*(GE) was doubled	(*108*)
Crude palm oil	Neutralisation (only the addition of alkali, refining step was not like industrial practice)	1 mmol/kg NaHCO_3_	*w*(3-MCPDE) and *w*(GE) reduced 81 and 84 %, respectively	(*5*)
Crude palm oil	Neutralisation	KOH and NaOH were used (other conditions not given)	*w*(3-MCPDE) reduced 35 % and 45 % when NaOH and KOH were used, respectively	(*20*)
Pre-refined palm oil	Bleaching	*w*(Tonsil Optimum 214 FF bleaching earth)=0.8 % was added to the oil (90 °C, up to 1 kPa) and removed by filtering after 20 min treatment	*w*(3-MCPDE and related ester) was reduced from 6.06 to 2.48 mg/kg (59 % reduction)	(*89*)
Peanut oil (pressed)	Bleaching	The degummed oil was preheated to 95 °C and bleached by adding activated clay (activated by 3 % hydrochloric acid) up to 1 kPa and then filtered after 25 min treatment	*w*(3-MCPDE) was reduced by approx. 15–22 % after bleaching. Although DGF standard method C-VI 18 (10) of the German Society for Fat Science was used, GE results were not given	(*37*)
Crude soybean oil	Degumming, neutralisation, bleaching and deodorisation	Use of acid in the degumming stage and acid-activated clay	*w*(3-MCPDE) increased compared to crude oil	(*109*)
Crude palm oil	Degumming	Acid degumming (0.06 % of 85 % H_3_PO_4_ for 20 min at 90 °C) and water degumming (1 % water, 10 min at 90 °C)	Reduction ranging from 56 to 78 % for *w*(3-MCPDE) and 49–56 % for *w*(GE)	(*110*)
Rice bran oil and palm oil	Bleaching	20 g oil was bleached with 200 mg activated earth at 110 °C and 10 kPa, after 20 min, cooled to 30 °C and filtered	*w*(GE) of the model oil spiked with different ester forms of glycidol was reduced effectively to ˃0.1 mg/kg	(*72*)
Crude palm oil	Bleaching	At 105 °C for 20 min with acid-activated bleaching earth (1, 2 and 3 %)	*w*(3-MCPDE) reduced as bleaching earth content increased	(*38*)
Crude palm oil	Bleaching	At 95 °C for 20 min with *w*(clay)=0.5 %	The use of low Cl bleaching earth resulted in lower *w*(MCPD) than the use of high Cl bleaching earth	(*111*)
Crude palm oil	Bleaching	*w*(pure-Flo B80 natural bleaching earth)=10 %	Bleaching reduced 93 % of *w*(GE) formed during deodorisation, while *w*(3-MCPDE) were unaffected	(*29*)
Palm oil	Deodorisation	10 g sample was heated to 200–290 °C for 45–360 min; 20 kg/t steam was purged	Despite the increase in deodorisation temperature to 270 °C, the 3-MCPDE+GE content in the samples increased. Deodorisation for 240-360 min at 290 °C resulted in a decrease in contaminant levels	(*20*)
Peanut oil (pressed)	Deodorisation	At 220-260 °C up to 3 h under 200 Pa with steam speed of 0.1 g/min	*w*(3-MCPDE and related matter) were reduced after 2 h of deodorisation	(*37*)
Crude palm oil	Bleaching	*w*(zeolite-Fe)=1.5–15 % and different bleaching temperature (60–140 °C)	The most effective bleaching procedure was achieved with a *w*(zeolite-Fe)=5 % and a bleaching temperature of 80 °C for 30 min	(*112*)
Palm oil	Bleaching	Using activated carbon treated with a 2 M HCl solution	*w*(3-MCPD) 80 % reduction and *w*(GE) 97 % reduction	(*113*)
Palm oil	Bleaching	Using metal-organic frameworks (MOFs)	The MOFs, which are composed of 2,6-naphthalenedicarboxylic acid (Fe-MIL-88_BDC), exhibited the most effective adsorption capabilities	(*114*)
Palm oil	Bleaching	Using zeolite	The use of beta zeolites led to the most effective removal of 3-MCPDE (86 %) compared to other zeolites tested. Using a synergistic blend of beta zeolite and AC, the efficiency of removing 3-MCPDE was significantly improved to 94 %, resulting in a simultaneous reduction of 75 % in GE	(*115*)
Coconut oil	Bleaching	At 105 °C with *w*(activated clay)=1 %. Bleaching was performed under vacuum. After 30 min, the slurry was allowed to cool (to 50 °C) and filtered	In crude coconut oil *w*(3-MCPDE) and *w*(GE) were 0.21 and 0.29 mg/kg, respectively. After bleaching, *w*(3-MCPDE) remained the same, but *w*(GE) was reduced to 0.19 mg/kg	(*116*)
Camellia oil	Degumming, bleaching and deodorisation	Degumming water addition at 2.97 %, bleaching earth (acid activated) 2.69 %, deodorisation temperature 230 °C, deodorisation duration 90 min	*w*(3-MCPDE) reduced by 76.9 %	(*116*)
Palm olein oil	Bleaching and deodorisation	Bleaching; 95 °C up to 1 h under 8 kPa using neutral and acid-activated bleaching earth Deodorisation (at 200–230 °C up to 1.5 h under 0.15–0.2 kPa)	Use of neutral bleaching earth and deodorisation at 230 °C led to *w*(MCPD) reduction of 69.91 %. The glycidol esters may be effectively reduced by up to 93.85 % with the use of acid-activated bleaching earth and deodorisation at a temperature of 230 °C, without the need for neutralisation	(*117*)
Palm oil	Deodorisation	At 250 °C up to 2 h using 100 g bleached oil	Potassium acetate reduced *w*(3-MCPDE) almost 99 % and *w*(GE) 40 %. Sodium hydrogencarbonate, sodium acetate and sodium carbonate were able to lower *w*(3-MCPDE) by more than 80 %	(*118*)
Palm oil	Deodorisation	Different temperatures (210–270 °C) and time (30–120 min)	After 30 min at 210 °C, the formation of 3-MCPDE was most pronounced under the mildest conditions	(*119*)
High oleic sunflower and rapeseed oil	Water washing, degumming, bleaching and deodorisation	Different deodorisation temperatures (220–260 °C), at 70 °C up to 20 min using 0.5 % citric acid for degumming, at 95 °C up to 20 min with 3 different bleaching earth (0.1–2 %)	Double refining successfully decreased *w*(GE) in high oleic (HO) sunflower and rapeseed oil by 70 and 94 %, respectively	(*120*)

Washing the oil prior to degumming was reported as method of eliminating free chloride ions to reduce the formation of 2- and 3-MCPDE. Pre-washing with 10 % deionised water at 70 °C for 20 min resulted in a reduction of the final 2- and 3-MCPDE content by 19 and 22 %, respectively (*109*).

Gums, mainly a mixture of hydratable and non-hydratable phospholipids, are removed in the initial step of the refining process (*75*). The degumming step is generally applied in both physical and chemical refining by the addition of phosphoric acid to water. During refining, the degumming step can slightly reduce the 3-MCPDE content of crude and pre-refined palm oil by partly removing potential precursors such as diglycerides, monoglycerides, free chloride ions and polar compounds relative to triglycerides through their dissolution and removal with water and phosphoric acid (*37*, *89*). On the contrary, in another study, after the degumming step, the 3-MCPDE content of hot-pressed peanut oil was either not affected or it slightly increased from 0.13 to 0.14 mg/kg (*37*). The lower amount of phosphoric acid added during degumming increases the reduction of 3-MCPDE because an acidic medium induces the formation of 3-MCPDE and GE through a nucleophilic substitution pathway or the formation of intermediate acyloxonium ions (*65*, *109*). Although the degumming temperature was not reported by Sim *et al.* (*38*) as an effective factor in the formation of 3-MCPDE and GE, increasing the degumming temperature to 105 °C increases the solubility of phospholipids in the oil, leading to a decrease in degumming efficiency and, consequently, in a potentially higher 3-MCPDE formation.

Neutralisation or alkaline refining refers to the removal of free fatty acids by chemical saponification using an alkaline solution. Neutralisation, a step of chemical refining, contributes less to the formation of 3-MCPDE than bleaching and deodorisation (*109*). Franke *et al.* (*89*) reported an increase of 0.91 and 0.09 mg/kg in 3-MCPDE mass fractions of prerefined palm oil and crude palm oil, respectively, which was still lower than the increase during deodorisation. On the contrary, chemical neutralisation of organic palm oil with 2 L of 33 % NaOH solution at 70 °C for 20 min and subsequent washing led to a 43 % decrease in 3-MCPDE mass fraction. However, the mitigation effect was not observed for GE, where neutralisation increased the GE mass fraction up to 29.4 mg/kg, while the GE mass fraction of physically refined (control) samples was 10.90 mg/kg (*108*). Neutralisation with sodium carbonate and sodium hydrogencarbonate had completely different effects on the GE mass fractions in crude palm oil. The GE was eliminated almost completely by neutralisation with sodium carbonate, while the amount of 3-MCPDE was reduced. When 1 mmol/kg sodium hydrogencarbonate was used, the total ester mass fraction was reduced to 1 mg/kg (*5*). In addition, Pudel *et al.* (*20*) reported that neutralisation caused a reduction in 3-MCPDE and related compounds when compared to crude palm oil. Moreover, when palm oil was neutralised with potassium hydroxide, a 45 % lower content of 3-MCPDE and related compounds was formed, while the reduction was 35 % when sodium hydroxide was used (*20*). The conflicting results of the reported effects of neutralisation on the content of 3-MCPDE and GE may be due to the use of washing before or after neutralisation, which reduces the concentration of precursors and ions.

Bleaching is the step in which pigments are removed using acid-activated or natural bleaching earth, which are adsorptive materials for pigments and phospholipids *via* van der Waals forces (*75*). Since bleaching clay adsorbes polar groups, numerous studies have reported a reduction of ester content during bleaching. Franke *et al.* (*89*) found a 59 % reduction of 3-MCPD. However, their analytical method at that time could not distinguish between 3-MCPDE and GE, so it was unclear which of the two was responsible for the reduction. Li *et al.* (*37*) conducted a separate analysis of 3-MCPDE and GE content. They reported a reduction of 15 to 22 % 3-MCPDE content after bleaching hot-pressed peanut oil with 3 % acid-activated bleaching earth. Another study focused on the effects of chemical refining steps on the physicochemical properties of coconut oil and the changes in 3-MCPDE and GE content of the samples (*121*). The GE mass fraction decreased from 0.29 to 0.21 mg/kg, which was statistically significant, however, the 3-MCPDE content was not significantly reduced. Sim *et. al.* (*38*) investigated the effects of refining conditions on the 3-MCPDE and GE content of crude palm oil using an optimisation approach of the response surface methodology. As the experimental design varied the amount of phosphoric acid, degumming temperature, bleaching earth amount and deodorisation temperature, the isolated effect of bleaching condition was not reported. However, linear modelling showed that a higher content of acid-activated bleaching earth decreased the 3-MCPDE mass fraction. Since the GE mass fraction varied between 0 and 0.64 mg/kg, a valid model for GE could not be determined. Furthermore, researchers indicated that the application of a modified (optimised) refining process enabled the extraction of refined palm oil with a lower 3-MCPDE mass fractions from 1.10 mg/kg (conventionally refined) to around 0.10 to 0.79 mg/kg, with a total reduction of 90 %. For mass fraction of 0.85 mg/kg GE, a reduction of 24.7 to 65.0 % was achieved with conventional refining (*38*). Some studies indicated a great potential of bleaching for the reduction of GE content in oil. The use of acid-activated bleaching earth was an undesirable practice because of the reported 3-MCPDE and GE propagation effect of chloride ion groups formed during acid activation of bleaching earth (*111*). However, Shimizu *et al.* (*72*) indicated that bleaching caused a significant reduction in the GE content in model oils spiked with different esterified forms of glycidol (palmitate, stearate, oleate, linoleate and linolenate). The mass fractions were reduced below 0.1 mg/kg regardless of the spike amounts. Studies have also indicated that bleaching was a crucial step in reducing GE mass fractions. Oey *et al.* (*122*) pointed out that bleaching reduced 93 % of the GE content formed during deodorisation, while the 3-MCPDE content remained unaffected. Anis *et al.* (*112*) used zeolite-Fe to improve the bleaching process and decrease the chlorine concentration, and optimised bleaching settings by the use of response surface methodology (RSM). They also evaluated the properties of the bleached crude palm oil. Bleaching duration, zeolite-Fe content and bleaching temperature were evaluated as factors and subsequently adjusted. The results showed that the concentration of Fe in the modified zeolite-Fe increased significantly by 71.89 % compared to the natural zeolite. The mass fraction of zeolite-Fe, the duration of bleaching and the temperature of bleaching all had a remarkable effect on the adsorption of chlorine. The most effective bleaching process was achieved with a zeolite-Fe mass fraction of 5 % and a bleaching temperature of 80 °C for a duration of 30 min. The adsorption capacity of pretreated activated carbon for 3-MCPDE was evaluated in a batch system at different adsorption temperatures (*112*). Restiawaty *et al.* (*113*) found that the mass fraction of 3-MCPDE in the refined, bleached and deodorised palm oil was effectively reduced by 80 %, from 19 700 to below 4000 mg/kg, using activated carbon treated with a 2 M HCl solution. The application of the identical therapy led to a significant decrease in GE from 6680 to 200 mg/kg, which corresponds to a reduction of up to 97 % (*113*). In a study by Ahn *et al.* (*114*), metal-organic frameworks (MOFs) were used to selectively adsorb and remove 3-MCPD and glycidol, which are known to be potential genotoxic carcinogens. To increase the efficiency of adsorption, a series of Fe-MIL-88 compounds with various ligand types were employed, followed by further modification by successive carboxylation and deprotonation procedures. Of these ligands, the MOFs, consisting of 2,6-naphthalenedicarboxylic acid (Fe-MIL-88_BDC), exhibited the most effective adsorption capabilities. Surface modification led to a significant increase in the efficiency of 3-MCPD and glycidol. Although it has lower porosity, the deprotonated Fe-MIL-88_BDC showed the maximum adsorption capacity among the mentioned adsorbents. The researchers discovered that the significant increase in adsorption efficiency was a result of esterification with carboxyl groups on 3-MCPD and glycidol. The adsorption performance was found to be exclusively in the 3-MCPDE and GE forms (which are precursors of 3-MCPD and glycidol) in palm oil, without any change in the content of fat or minor components (*114*).

In another study, Restiawaty *et al.* (*115*) investigated the reduction of the 3-MCPDE and GE content in refined, bleached and deodorised palm oil by utilizing zeolite as an adsorbent. The adsorption was carried out in a batch reactor, varying the adsorbent percentage, temperature, zeolite type and the combination of zeolite and activated carbon. The results show that the optimum temperature for adsorption was 35 °C when 2 % zeolite was used. The use of beta zeolites led to the most effective removal of 3-MCPDE (86 %) compared to other zeolites tested. This can be attributed to their large pore volume, Si/Al ratio and overall acidity. By using a synergistic mixture of beta zeolite and activated carbon, the efficiency of 3-MCPDE removal was significantly improved to 94 %, resulting in a simultaneous reduction of GE by 75 % (*115*).

Deodorisation is the final step in which the main aim is to remove the odour-forming material from vegetable oil by distillation. As the previous neutralisation step is not used in physical refining, the free fatty acid content is also removed at this point. Significant amount of GE and 3-MCPDE formed during deodorisation as higher temperatures (>220 °C) are removed in this step. An earlier study indicated that the content of 3-MCPDE and a related compound (GE) decreased at extreme temperatures (290 °C) during deodorisation for 240–360 min, although it increased 10 times when deodorized at 270 °C. It was also reported that the amount of contaminants increased with time up to 250 °C, particularly the amount of GE. This effect was reversed at temperatures above 270 °C, and at 290 °C the amount of contaminants decreased with time, probably due to thermal degradation reactions or distillation (*20*). A similar effect of thermal degradation was also found in another study where 3-MCPDE and related matter content in hot-pressed peanut oil was reduced after deodorisation at 220–260 °C for 2 h (*37*). Since deodorisation accelerates the formation of 3-MCPDE and GE, some optimisation studies have been carried out to limit or minimise the formation of 3-MCPDE and GE during deodorisation while meeting the industrial demand for refined oil. The optimisation studies aim to find the factors and their interactions that have an effect on the formation of 3-MCPDE and GE during processes. Studies suggest that temperature is the most significant factor in the formation of 3-MCPDE and GE during deodorisation, and that duration is another important factor as it represents effective total thermal load of the oil (*110*). Sim *et al.* (*38*) investigated the effect of optimised refining conditions including phosphoric acid amount, degumming temperature, bleaching earth amount and deodorisation temperature (240, 250 and 260 °C) and reported that the application of a lower temperature was important to limit the formation of 3-MCPDE and GE during deodorisation. During the optimisation of deodorisation parameters, while temperature was the most effective factor for 3-MCPDE and GE formation, the provoking effect of temperature on GE formation was not observed at high steam stripping rates. This result was associated with an increased mass transfer rate of precursors such as diglycerides and GE at high steam stripping rates (*92*). Optimisation studies generally provided valid results and approaches only for the used laboratory-scale equipment and experimental conditions discussed. Positive results were observed in the reduction of 3-MCPDE and GE in the final product by using the optimised refining conditions at pilot and industrial scale (*38*). Camellia oil is a type of oil that has a high 3-MCPDE content due to the deodorisation process applied at high temperatures, however, certain physical refining steps were optimised to minimise the 3-MCPDE content (*116*). A 76.9 % reduction of 3-MCPDE content was achieved compared to commercial samples. The study suggests degumming at 2.97 % moisture, degumming temperature of 50.5 °C, activated clay amount of 2.69 %, deodorisation temperature of 230 °C and deodorisation time of 90 min.

Elmas and Akpinar Bayizit (*117*) investigated the optimal process parameters to minimise the formation of 3-MCPD and GE during palm oil refining. They have varied refining parameters such as bleaching agent and deodorisation temperature, and obtained a reduction of 3-MCPD by 69.91 % with neutral bleaching earth and deodorisation at 230 °C, and a reduction of GE by 93.85 % with acid-activated bleaching earth and deodorisation at 230 °C without neutralisation.

Studies have also investigated the reduction of 3-MCPDE and GE by the use of adsorbents, metals, salts and amino acids before and during deodorisation (*118*). Potassium acetate reduced the 3-MCPDE content by almost 99 % and the GE content by 40 %. However, it led to an increase in free fatty acid content in the refined oil. The other compounds used caused a small decrease of just 1–30 % of 3-MCPDE and 6–49 % of GE. In the same study, other salts such as sodium hydrogen carbonate, sodium acetate and sodium carbonate were able to reduce 3-MCPDE by more than 80 %. However, they caused an increase in GE and changes of both the colour and FFA content of the refined, bleached and deodorised palm oil. Similar results were obtained when adsorbents, metals or amino acids were used, indicating their effectiveness in removing a specific pollutant, either 3-MCPDE or GE.

Furthermore, Tivanello *et al.* (*119*) investigated the deodorisation of palm oil at different temperatures (210, 230, 250 and 270 °C) and durations (30, 60, 90 and 120 min), and concluded that the formation of 3-MCPDE was most pronounced under the mildest conditions (30 min at 210 °C), with values ranging from 1.91 to 2.70 mg/kg. Notably, the study did not find a significant correlation between the 3-MCPDE content and the physicochemical changes in the deodorised palm oil (*119*).

Mayayo *et al.* (*120*) carried out studies with the aim of developing strategies to reduce the formation of contaminants, especially GE and 2- and 3-MCPDE in organic oils. The focus was on sunflower oil with high oleic content and rapeseed oil, and the research looked specifically at the refining process to achieve this reduction. The study compared several techniques, including twofold refining with a high or low deodorisation temperature, water washing and increasing amounts of bleaching earth, with a single physical refining treatment (control treatment). The results showed that the twofold refining successfully reduced the GE content in sunflower oil with high oleic content and rapeseed oil by 70 and 94 % respectively, compared to the control treatment. The second degumming and bleaching steps were very important for the removal of GE contamination. Both 2- and 3-MCPDE were formed during the temperature ramping phase before deodorisation and their content remained constant throughout the refining process. Increasing the amount of bleaching earth by a factor of two led to a 48 % reduction in the content of 2- and 3-MCPDE in the sunflower oil with high oleic content. However, no reduction was observed in rapeseed oil (*120*).

### Effects of the oil modification on the reduction of 3-MCPDE and GE

In addition to the refining processes, several modifications are used to produce different fat and oil products to alter the physical properties of the fats and oils to meet the requirements of the intended use. The processes that offer the possibility of modifying fats and oils are fractionation, winterisation, interesterification and hydrogenation (*7*, *123*). Fractionation is a common method applied to palm oil that often refers to drying; however, a wet or solvent fractionation can also be used. Wet fractionation, typically applied to cottonseed products, uses hexane or acetone to separate different components in the miscella stage. Kyselka *et al.* (*98*) investigated the reduction of 3-MCPDE and GE content in palm oil during hydrogenation and wet fractionation. By wet fractionation with acetone palm oil was fractionated into four different fractions, namely super stearin, hard stearin, super olein and top olein. The results show that the distribution of 3-MCPDE and GE in the fractions tends to be more than a reduction. The 3-MCPDE and GE were highly concentrated in the top olein phase at 16.16 and 9.2 mg/kg, respectively. Super olein contained 5.01 mg/kg 3-MCPDE and 1.08 mg/kg GE. Super stearin and hard stearin contained lower amounts of 3-MCPDE and GE. The results showed that 3-MCPDE and GE tend to remain in the liquid fractions of palm oil during fractionation. During the complete hydrogenation of the palm oil sample, the mass fraction of 3-MCPDE and GE was reduced from 4.72 to 2.17 mg/kg and from 1.07 to 0.62 mg/kg, respectively. Although deodorisation applied after complete hydrogenation caused a slight increase in the contents of both contaminants, the reductions during hydrogenation were notable. Another significant decrease in glycidyl ester content was observed after bleaching of fully hydrogenated palm oil, where GE content was not detected, while 3-MCPDE content was not affected by the bleaching (*98*).

Enzymatic approaches have been shown to be a promising tool for reducing 3-MCPD in the extraction of palm oil, serving as an alternative to mechanical extraction. Modification of the fatty acid arrangement in the final product can be customised by the development of lipases used in the enzymatic production of structural lipids to benefit specific initial characteristics of materials, such as the location of the glycerol backbone and fatty acids. In 2010, Bornscheuer and Hesseler (*124*) investigated the potential of using enzymes to remove 3-MCPDE and GE from refined oils. They were the pioneers in uncovering the fact that 3-MCPDE can be converted into the harmless by-product glycerol by enzymatic cascade reactions. The enzyme cascade consisting of an epoxide hydrolase from *Agrobacterium radiobacter* AD1 and a halohydrin dehalogenase from *Arthrobacter* sp. AD2 was used to fully convert 3-MCPD into glycerol. This conversion took place at a lower reaction temperature of 30 °C in a thermomixer (*124*). Bel-Rhlid *et al.* (*125*) conducted another enzymatic treatment with 1242 and 0.3358 mg/kg of racemic 3-MCPD in the presence of oxygen. At pH=8.2 and in the presence of glucose, a mass fraction of 1242 mg/kg of racemic 3-MCPD was converted at an ideal rate of 68 %. In contrast, without glucose, a mass fraction of 0.3358 mg/kg of 3-MCPD degraded by 73 % after 72 h (*125*). In addition, Tsai *et al.* (*126*) used modified QuEChERS (quick, easy, cheap, effective, rugged and safe) and lipase hydrolysis as pretreatment methods for sample material to detect 3-MCPDE in edible oils. Acetonitrile and salts are used in QuEChERS, a common sample preparation method for the extraction of highly polar compounds such as 3-MCPD. Their approach showed superior precision and accuracy compared to the widely used official method (AOCS Cd 29c-13), as they presented a rapid and accurate analytical method to quantify the amount of 3-MCPDE in edible oils (*126*). In addition, Miyazaki and Koyama (*127*) conducted a study that used *Candida cylindracea* in an indirect enzymatic technique to identify 3-MCPDE. This method involved the removal of sodium bromide during the hydrolysis. Recovery rates of 3-MCPD from concentrated sardine oil were found to be good, ranging from 91 to 109 % at a mass fraction of 20 mg/kg (*127*). In addition, Chai *et al.* (*128*) investigated the effect of tallow by enzymatic hydrolysis and the addition of NaCl on the production of 3-MCPDE. In the preparation of beef flavour, they found that the specific conditions of enzymatic hydrolysis (80 % tallow (*m*/*V*), 47.5 °C, pH=7 and 9.5 h) had a notable effect on the formation of 3-MCPD mono- and diesters. There were no appreciable amounts of 3-MCPD monoesters detected, while the amount of 3-MCPD diesters at all temperatures depended strongly on the content of NaCl and the time of its addition (either before or after the thermal reaction). The reason for this is that tallow has a very low melting point of 47.5 °C. At temperatures above 50 °C, the lipase becomes practically inactive (*128*). According to all published studies, the decrease in the synthesis of 3-MCPDE can be attributed to the elimination of the associated precursors before deodorisation. This is because several studies have shown that most 3-MCPDE are formed during the deodorisation of oil. Research into the exact processes behind the formation of 3-MCPD is still ongoing. In order to effectively reduce and eliminate 3-MCPD from vegetable oils by enzymatic hydrolysis, it is crucial to understand the link between the potential of the enzyme and its contribution to the process.

Deep fat frying is known as a cooking method that potentially triggers the formation of 3-MCPDE and GE due to the prolonged exposure of the oil to high temperature. The content of both contaminants was reduced after three days of frying; however, 3-MCPDE and GE formation in rosemary oleoresin and *tert*-butylhydroquinone (TBHQ) decreased to a larger extent in refined, bleached and deodorised palm oil fortified with 200 mg/kg natural (rosemary and sage extract) and synthetic (butylated hydroxy tolune (BHT), butylated hydroxy anisole (BHA) and TBHQ) antioxidants, from 3.2 to 1 mg/kg and from 5.2 to 3.5 mg/kg, respectively (*14*). The reduction of 3-MCPDE and GE was associated with the reducing effect of antioxidants on the intermediate radical acyloxonium shortly before the nucleophilic attack of chloride ions during the 3-MCPDE production pathway (*14**,**129*).

As the theory about the inhibitory effect of antioxidants on the formation of 3-MCPDE and GE was established, the potential of natural antioxidants to replace unsafe conventional synthetic antioxidants was investigated. Therefore, sesame oil was used to inhibit the formation of GE in corn oil, palm oil and rice bran oil during the deodorisation. Sesame oil contains a considerable mass fraction of sesamin, sesamolin and sesamol lignans (6.5–17.3 mg/kg), and tocopherols (56.9–99.3 mg/kg, mainly α-tocopherol), which all have antioxidant activity. When the addition of sesame oil was increased from 10 to 50 %, the inhibition of GE formation increased from 4.29 to 18.86 % in corn oil, from 16.82 to 27.39 % in palm oil, and from 23.64 to 29.89 % in rice bran oil (*130*).

A recent study investigated the inhibitory effect of seven antioxidants, including quercetin, astaxanthin, TBHQ, propyl gallate, l-ascorbyl palmitate, puerarin and α-tocopherol, on the formation of GE in rice bran oil during the exposure to high temperature (*131*). Among the seven antioxidants, propyl gallate, astaxanthin and l-ascorbyl palmitate with different antioxidant mechanisms had a better inhibitory effect on GE formation. The study also reported that after high temperature application at 240 °C for 1.5 h, the inhibition rates decreased due to probable degradation of the antioxidants. In particular, the epoxy structure of GE would compromise their stability at high temperatures, leading to thermal degradation to MAG *via* a ring-opened process. The increasing trend in the inhibition rates of the antioxidants was attributed to the combined suppressive effect of antioxidants on GE formation and thermal degradation of GE during high temperature application.

## DEVELOPMENT OF METHODS FOR THE EXTRACTION AND DETERMINATION OF 3-MCPDE AND GE

Chromatographic techniques are widely used today in studies for the detection of chloropropanols or their esters in food matrices. Apart from these methods, electrochemical and optical methods are also used. Each technique offers different mechanisms for qualitative or quantitative analysis. Intrinsic limitations such as molecular structure, low volatility and low molecular mass hinder the direct gas chromatographic (GC) analysis of chloropropanols. Researchers addressed these challenges with a pretreatment step involving derivatisation. This technique converts chloropropanols into more volatile derivatives, minimising unwanted interactions with other sample components during preparation and GC analysis. Derivatisation has enabled the development of different analytical methods for the determination of chloropropanols, including GC (*132*), GC-MS (*133*) and HPLC-FLD (*134*). When analysing chloropropanol, the choice of derivatisation reagent is crucial. Phenylboronic acid (PBA) (*135*), butaneboronic acid (BBA) (*136*, *137*), heptafluorobutyrylimidazole (HFBI) and heptafluorobutyric acid (HFBA) (*138*) are common reagents for the detection of chloropropanol. However, the innate structural properties of chloropropanols can lead to adverse interactions with the GC system, which can affect sensitivity and cause poor peak shape. It is also problematic to identify targets based on retention times alone.

GC-MS offers superior sensitivity for the determination of chloropropanols and achieves LODs below 3–5 μg/kg for 3-MCPD in various food products (*139*). The Association of Official Analytical Chemists (AOAC) has established a GC-MS method for the analysis of 3-MCPD in food with an LOD of 0.005 mg/kg (*140*). HPLC-FLD is an alternative, especially because of its high sensitivity. Prior to HPLC-FLD analysis, Hu *et al.* (*141*) developed a derivatisation method using periodate oxidation for the detection of 3-MCPD in vegetable oil and water, and achieved an LOD of 0.36 ng/mL. This method is based on the oxidation cleavage of 3-MCPD with sodium periodate to generate chloroacetaldehyde. Subsequent fluorescence derivatisation with adenine enables HPLC analysis with FLD detection. Compared to GC-MS, HPLC-FLD has a significantly lower LOD (by approx. ten orders of magnitude) and eliminates the need for complex pretreatment, extraction or enrichment steps. Notably, in a relevant study, an average recovery of 95.36 % and a relative standard deviation (RSD) of less than 3.44 % was found for repeated measurements, highlighting the excellent accuracy and sensitivity of the method. Beyond derivatisation, researchers have explored alternative pretreatment methods for the detection of chloropropanols. These include molecularly imprinted polymer membrane extraction coupled with GC-MS (*142*) and the combination of ultra-high performance liquid chromatography (UHPLC) with microwave-assisted derivatisation (MAD) followed by HPLC-UV detection (*143*). These approaches offer versatility and can be used for a wide range of samples. They also achieve sufficient sensitivity for detection in the mg/kg or μg/kg range. However, there are also limitations. The techniques often require expensive equipment and time-consuming procedures. Furthermore, the presence of nucleophilic compounds in the extract can react with derivatisation reagents, resulting in increased background noise, compromised selectivity and reduced abundance of target ions. Notably, HFBI, a popular derivatisation reagent, is very sensitive to moisture, which can hinder consistent derivatisation.

Electrochemical approaches represent an alternative strategy for the detection of 3-MCPD. Sun *et al.* (*144*) developed a sensor based on a glassy carbon electrode with deposited gold nanoparticles (AuNP/GCE) and coated with a molecularly imprinted polymer film. This sensor showed high sensitivity (LOD of 3.8·10^-18^ mol/L) and excellent recovery rates (95.0–106.4 %) for the detection of 3-MCPD in soy sauce, demonstrating strong adsorption and selectivity. However, the complex manufacturing process and limited reusability of the sensor limit its practical application (*144*). Yuan *et al.* (*145*) proposed a method in which hemoglobin (Hb) is immobilised on magnetic molecularly imprinted nanoparticles. This approach uses the reducibility of Hb for the detection of 3-MCPD and achieves an LOD of 0.25 mg/L. The method relies on the electrochemically generated reduction peak current at -0.236 V, which is further reduced by 3-MCPD through Hb catalysis. Higher 3-MCPD amounts lead to a corresponding increase in the intensity of the reduction peak. While this method offers acceptable repeatability (RSD of 2.9 %), limitations include the need for nitrogen supplementation in the electrolyte and the short shelf-life of the modified electrode (*145*).

Fang *et al.* (*146*) introduced a fluorescence-based detection method for 3-MCPD in soy sauce using a molecularly imprinted polymer on a paper substrate. This method eliminates the need for pretreatment and enables direct sample analysis. With an LOD of 0.6 ng/mL, recovery rates between 97.2 and 105.3 % and an RSD below 5.6 %, it surpasses the accuracy of the HPLC-FLD approach. Notably, the HPLC-FLD method suffers from potentially incomplete conversion during the complex pretreatment steps, which include oxidation cleavage and derivatisation, as evidenced by the reported conversion rate of 98.96 %. However, the fluorescence method has its own limitations, including the fragility of the paper substrate and the lengthy surface modification. In addition to the methods explored here, several other techniques, such as UV-Vis spectroscopy, have been developed for the detection of chloropropanols, each offering unique advantages and disadvantages (*147*).

Traditional indirect methods remain the mainstay for the determination of chloropropanols and chloropropanol esters. This dominance can be attributed to several factors: affordability and accessibility of consumables and instruments, ease of use and repeatability, and satisfactory analytical performance (accuracy, precision and sensitivity). These methods typically involve a sequence of steps: alkaline or acid-catalysed cleavage, solid-phase extraction (SPE), derivatisation with PBA or HFBI, and final detection by GC-MS or GC-MS/MS. Future advances are likely to refine this core approach. Direct methods, while potentially valuable, are less commonly used due to the high cost of instrumentation and extensive standard requirements. Among indirect methods, lipase-catalysed cleavage, advocated by the AOCS, is a promising alternative with several advantages. However, further research and validation for different sample types are required for wider application. Solid-phase extraction (SPE) offers potential for improvement by minimising eluent volume and toxicity. Ideally, specialised SPE materials and columns tailored for efficient chloropropanol extraction should be developed. Non-derivatisation methods are promising replacements for derivatisation-based approaches as they offer less manipulation and lower consumption of resources. However, overcoming the challenges of chromatographic separation of chloropropanols remains critical. Rapid screening methods would benefit from simplified material preparation and streamlined determination procedures.

Custodio-Mendoza *et al.* (*43*) developed and validated two new indirect approaches for total 3-MCPD. 3-MCPD was released from 3-MCPDE by both alkaline and acidic transesterification processes. For the first time, these reactions were accelerated with ultrasound. The overall content of 3-MCPD was determined using an ultrasound-assisted dispersive liquid-liquid microextraction method and subsequently analysed by GC-MS. Both methods were optimised, with the emphasis on variables such as sample size, reagent quantity, temperature and agitation technique. The results showed that the alkaline approach achieved a lower limit of quantification, while the acidic method showed good accuracy and precision. Both approaches showed similar performance in terms of analytical properties. The alkaline method was shown to require fewer different types of aqueous solutions, lower amounts of organic liquids, less waste, less reaction time and lower temperatures. Ultrasound greatly shortens response times, resulting in faster, more environmentally friendly and more economical processes (*43*).

In conclusion, traditional indirect methods currently dominate the analysis of chloropropanols. While existing techniques are being further developed and new approaches explored, further research is needed to overcome limitations and improve overall analytical capabilities.

## CONCLUSIONS

3-Monochloropropane-1,2-diol (3-MCPD) and glycidyl esters (GE) are process contaminants found in vegetable oils and/or oil-containing food as a result of processing. While mono- and diglycerides and chloride ions are effective in the formation of these contaminants, high temperature and time are also important factors that increase their formation and quantity. Mono- and diglycerides formed during the breakdown of triglycerides can turn into glycidyl esters with genotoxic and carcinogenic properties at high temperatures and 3-MCPD esters with carcinogenic properties in the presence of chloride ions in the environment. According to the report published by EFSA in 2018, the tolerable daily intake of 3-MCPD was set at 2 µg/kg body mass. These contaminants are even found in different foods such as coffee, smoked foods and baby food. For this reason, the amount of 3-MCPD found in many foods and the reasons for its formation have gained attention in recent years. Existing studies to reduce or avoid these contaminants, improvement of process conditions, reduction of salt content of foods, reduction of mono- and diglyceride content in raw materials, deactivation of lipase enzyme, improvement of the growth and storage conditions of raw material, low temperature applications during refining, *etc.* are addressing these issues. Due to the negative impact of these toxic contaminants on consumer health, it is important to investigate them in more detail and carry out the necessary studies to reduce/prevent their formation and implement this into industrial processes. The scope and performance of direct techniques will be greately enhanced by the commercial availability of a solution with internal standards and mixed chloropropanol ester standards. Finally, as a quick screening method, less complicated material preparation and a quicker decision process are needed for alternative approaches. Future studies on chloropropanols, their esters and GE could focus on creating more practical experimental models to understand how they are formed, developing simpler sample preparation techniques, improving test methods with lower limits of detection, and finding more effective and economical ways to eliminate them.
